# Evaluation of skin reactions during proton beam radiotherapy – Patient-reported versus clinician-reported

**DOI:** 10.1016/j.tipsro.2021.05.001

**Published:** 2021-06-19

**Authors:** Marie-Louise Möllerberg, Ulrica Langegård, Birgitta Johansson, Emma Ohlsson-Nevo, Per Fransson, Karin Ahlberg, Petra Witt-Nyström, Katarina Sjövall

**Affiliations:** a RI.SE (Research Institute of Sweden), Scheelevägen 17, SE-223 70 Lund, Sweden; b Institute of Health and Care Sciences, Sahlgrenska Academy, University of Gothenburg, Sweden; c Experimental Oncology, Department of Immunology, Genetics and Pathology, Uppsala University, Uppsala University Hospital, Sweden; d University Healthcare Research Centre, Faculty of Medicine and Health, Örebro University, Sweden; e Department of Nursing, Umeå University, and Department of Cancercentrum, Norrlands University Hospital, Umeå, Sweden; f Danish Centre for Particle Therapy, Aarhus, Palle Juul-Jensens Boulevard 25, DK-8200 Aarhus, Denmark; g Faculty of Health Sciences, Kristianstad University, SE-291 88 Kristianstad, Sweden

**Keywords:** Clinician-reported, Patient-reported outcome, Primary brain tumour, Proton therapy, Radiotherapy, Skin reaction

## Abstract

**Background:**

Skin reaction is a common side-effect of radiotherapy and often only assessed as clinician-reported outcome (CRO). The aim was to examine and compare patient-reported outcome (PRO) of skin reactions with CRO for signs of acute skin reactions for patients with primary brain tumour receiving proton beam radiotherapy (PBT). A further aim was to explore patients’ experiences of the skin reactions.

**Methods:**

Acute skin reactions were assessed one week after start of treatment, mid-treatment and end of treatment among 253 patients with primary brain tumour who underwent PBT. PRO skin reactions were assessed with the RSAS and CRO according to the RTOG scale. Fleiss’ kappa was performed to measure the inter-rater agreement of the assessments of skin reactions.

**Results:**

The results showed a discrepancy between PRO and CRO acute skin reactions. Radiation dose was associated with increased skin reactions, but no correlations were seen for age, gender, education, occupation, other treatment or smoking. There was a poor agreement between patients and clinicians (κ = −0.016) one week after the start of PBT, poor (κ = −0.045) to (κ = 0.396) moderate agreement at mid treatment and poor (κ = −0.010) to (κ = 0.296) moderate agreement at end of treatment. Generally, patients’ symptom distress toward skin reactions was low at all time points.

**Conclusion:**

The poor agreement between PRO and CRO shows that the patient needs to be involved in assessments of skin reactions for a more complete understanding of skin reactions due to PBT. This may also improve patient experience regarding involvement in their own care.

## Highlights

• Radiotherapy is used in the treatment for primary brain tumours.
• Radiotherapy causes acute and late toxicities where skin reactions are most common.
• The potential for misreporting of toxicity in specific cases may be significant.
• Oncologist-reported and patient-reported outcomes of skin reactions are crucial.
• A combination of PROs and oncologists’ assessments is most accurate.

## Introduction

Radiotherapy is one of the treatment options for primary brain tumours, either as a supplement to surgery or alone, and upon indication, in combination with systemic therapy [1]. Radiotherapy causes acute and late toxicities where skin reactions are among the most common [2], [3]. In most cases, symptoms are mild, but may also be accompanied by swelling, redness, pigmentation, ulceration of the skin, and are usually experienced by the patient as pain, warmth, burning, and itching of the skin [2], [4]. Further, skin reaction depends on a variety of factors such as dose, volume, concomitant treatment with chemotherapy and on individual factors as age, smoking, coexisting diseases, tumour site, and genetics [5], [6]. Skin reactions may cause physical and psychosocial discomfort, resulting in a negative impact on their quality of life [7]. In severe cases, radiation-induced wounds may require a reduced radiation dose [8]. Severe acute skin reactions are also associated with a higher risk of chronic radiation-induced skin changes (e.g. fibrosis, telangiectasia) [9].

Particles, including protons, have unique physical properties. They slow down and stop at a specific depth in the patient depending on the energy given to the particles, depositing the therapeutic radiation dose at the site of the tumour. Proton beam radiotherapy (PBT) may significantly reduce unwanted doses to surrounding healthy tissues and organs and thereby result in reduced risks for acute and late toxicity [2], [3], [4]. The physical properties of protons result in a significant general reduction of the integral dose delivered to the patient, which may lead to the misconception among non-specialists that also skin reactions may be reduced [10]. Protons do not have the same skin-sparing effects as their photon counterparts, due to the build-up effect of photons. However, modern proton delivery technique, pencile beam scanning (PBS), compared to the older passive scattering technique, has the potential for reducing skin doses to levels comparable to photons.

Prevalence of skin reactions has been reported to as high as 86% among patients with brain tumours [8] and 89% among all patients that received PBT [11]. In these studies, most of the patients had grade 1 (faint erythema) skin reactions, and very few had grade 3 (desquamation) or 4 (ulceration). Additionally, when comparing PBT and conventional photon radiotherapy among patients with breast cancer, previous research has found a significantly higher rate of grade 2 radiation dermatitis in patients receiving PBT [12].

Traditionally, clinician-reported outcomes (CROs) are used to assess skin reactions [13]. The most widely used scoring systems are the Common Terminology Criteria for Adverse Events (CTCAE) [14] and the Radiation Therapy Oncology Group (RTOG) [15]. These assessments are based on clinician evaluation, which may ignore the patients’ perspective. However, there is a growing interest toward collecting patient-reported outcomes (PROs) for various types of symptoms [16]. Different PROs have been developed specifically for skin reactions, the Skin Toxicity Assessment Tool [17] and Skin-dex-16 [18]. A more general tool to report the frequency and intensity of symptoms (including skin reactions) related to radiotherapy and the related distress is the Radiotherapy-Related Symptoms Assessment Scale (RSAS) [19].

Patients assess both objective and subjective symptoms while clinicians assess the objective signs. Skin reactions like warmth and swelling may be experienced by the patient before they are visible to the clinician. This complicates a direct comparison [20]. It is suggested that PROs should be prioritised over clinical assessment because PROs is more accurate provided that valid and reliable PROM are available [13]. Earlier studies have described low agreement rates between PROs and CROs reports [13], [21], [22], [23]. Overall, patients often reported higher rates of toxicity compared to clinicians’ reports [21] and also compared to photographs [13]. Hence, as a practitioner for consideration, the potential for misreporting toxicity in specific cases can be significant. [22], [23].

It seems relevant to ask: how comparable and interpretable are these different methods of assessments? Could PROs also become the primary means of scoring and assessing acute skin reactions and used as tools for intervention?

### Aim

This study examine and compare patient-reported outcome of skin reactions with clinician-reported for signs of acute skin reactions for patients with primary brain tumour receiving proton beam radiotherapy. A further aim was to explore patients’ symptom distress due to skin reactions.

## Method

A longitudinal study was adopted with a prospective design.

### Participants and setting

The study was conducted at the Skandion clinic, a national Swedish proton therapy facility, situated in Uppsala, Sweden, and managed jointly by the seven Swedish regions hosting university hospital radiotherapy departments. The participants were included in a multicentre prospective PBT protocol (NCT02797366) [24] and in the present study which is a part of the Proton Care Study [25] with the aim to assess PROs and experiences related to PBT. A consecutive sample of 341 patients treated according to the PBT protocol from August 2015 to February 2019 were invited, and 253 (74.2%) patients agreed to participate. Inclusion criteria were adult patients aged 18 years or older who were diagnosed with primary brain tumour, and able to read and speak in the Swedish language.

#### Treatment

All preparation (construction of individual immobilization equipment, CT and treatment planning) prior to PBT is conducted at one of the seven university clinics in Sweden and transferred to the proton clinic. The treatment is given daily, Monday through Friday over 4–7 weeks. After treatment completion, patients are referred to their university clinic for follow-up. It is common for the patients to have received another cancer treatment before the PBT, e.g. surgery, chemotherapy or both. The usual radiation dose for treatment of primary brain tumor is between 50 and 58 Gy but can vary from 30 to 66 Gy.

#### Procedure

Coordinators at the seven university hospitals were responsible for identifying eligible participants. Study information was provided orally via telephone and written information were sent to eligible participants. Participants who agreed to participate signed an informed consent. Patients’ reports could be completed as a paper or web-based questionnaire. Web-based questionnaires were sent by email. Patients who chose paper-based questionnaires were provided with prepaid envelopes and asked to post the questionnaire at the end of treatment. A reminder was sent if questionnaires were not returned within one week.

### Data collection

Patient and clinician reported skin reactions were collected one week after starting PBT (start PBT); three weeks after starting PBT (mid PBT); and at the end of PBT (end PBT).

#### Patient-reported signs and symptoms of skin reactions

The questionnaire consisted of demographic questions and the Radiotherapy-Related Symptoms Assessment Scale (RSAS) [19]. RSAS aims to measure symptom intensity (and thereby the frequency) and symptom distress. It consists of 13 items rating symptom frequency and intensity from grade 1 (not at all), 2 (little), 3 (a great deal) to grade 4 (very much); and symptom distress from grade 1 (no concern to me), 2 (little concern to me), 3 (much concern to me) to 4 (greatest concern to me). It is also possible to answer N/A (not applicable). RSAS was psychometrically evaluated for patients with primary brain tumours receiving PBT in Sweden. The results showed acceptable psychometric properties, including reliability, responsiveness, and validity [26]. The item used in this study to measure the grade of skin reactions was, “*Have you during the past day experienced any concern of the skin within the treated area?”*

#### Clinicians reported signs of skin reactions

Medical data and clinicians’ assessment of skin reactions of each patient were collected from patients’ medical records. At the Skandion clinic, the clinicians assess symptoms on a weekly basis during the treatment period including the use of the Radiation Therapy Oncology Group (RTOG/EORTC) grading system to evaluate patients’ symptoms [15]. The RTOG skin toxicity scale is rated on a categorical scale of 0 through 4 (0 = no reaction, 1 = slight erythema, 2 = bright erythema or patchy desquamation, 3 = confluent desquamation, or 4 = ulceration).

### Statistics

For RSAS those who answered N/A were interpreted and transformed to RSAS 1 (no symptoms and of no concern). Patients’ reported skin reactions were compared to clinicians’ assessments of skin reactions by analysing if intensity as assessed with RSAS corresponded to severity as assessed with RTOG, i.e. if RSAS 1 corresponded to RTOG 0, RSAS 2 to RTOG 1, RSAS 3 to RTOG 2, and finally RSAS 4 to RTOG 3-4. Since the current study specifically targeted acute skin reactions during the treatment period, there should be a small number of participants experiencing RTOG 3-4.

Descriptive statistics were calculated to describe demographic data and the frequency and intensity of acute skin reactions. Multiple linear regression (MLR) analyses were performed to determine whether skin reactions according to RSAS or RTOG, were associated with age, radiation dose (continuous variables), gender, education, occupation, other tumour treatment or smoking (dichotomous variables). The regression analyses were based on robust standard errors as the assumption of normality was violated. Multicollinearity was assessed with variance inflation factor (VIF). No problems with multicollinearity were observed between independent variables according to the variance inflation factor (VIF; mean = 1.77–1.85 for RSAS and RTOG at the three timepoints).

Fleiss’ kappa was performed to measure the inter-rater agreement of PROs and CROs assessments of skin reaction at the three time points [27]. Fleiss introduced a category-specific kappa score which is a coefficient of agreement between observers, correcting for the proportion of agreement that could have occurred by chance. A kappa score of 1 indicates perfect agreement, a kappa score of 0 indicates that the variation in agreement can be explained purely by chance, a negative kappa score indicates that the variation in agreement was less than expected by chance and a kappa score of – 1 indicates no observed agreement [27]. The kappa statistics were interpreted as values of > 0.8 excellent agreement, 0.6–0.8 suggested good agreement, 0.2–0.6 moderate agreement, and less than 0.2 demonstrated poor agreement [28].

The statistical significance level was set at p < 0.05. Data were analysed using SPSS statistics 26.0 (IBM Corp., Armonk, NY, USA).

## Results

### Patient characteristic

Of the 253 participants 107 (42.3%) completed the web-based questionnaire and 146 (57.7%) by paper. Participants’ mean age was 47.9 years (SD = 14.2) and 131/253 (51.8%) were female (Table 1).

**Table 1:** Demographic characteristics of patients with primary brain tumour who underwent proton beam radiotherapy (n = 253).

Variables	Participants
Age	Mean (SD) [Range]
	47,9 (14.2) [19–84]

Sex	n (%)
Male	122 (48.2)
Female	131 (51.8)

Education, n (%)	
Up to high school (12 years)	135 (53.4)
University	114 (45.1)
Missing	4 (1.6)

Occupation, n (%)	
Employed/sick leave	208 (82.2)
Retired	44 (17.4)
Missing	1 (0.4)

Smoker, n (%)	
Current smoker	35 (13.8)
Non-smoker (include both previous – and never smokers)	145 (57.3)
Missing	73 (28.9)

Other tumor treatment, n (%)	
Surgery	98 (38.7)
Chemotherapy	7 (2.8)
Surgery and Chemotherapy	65 (25.7)
Missing	83 (32.8)

Total radiation dose, n (%)	
30–49 Gy	15 (5.9)
50–58 Gy	206 (81.4)
59–66 Gy	32 (12.6)

#### Skin reactions at one week after start of PBT

**Fig. 1:**
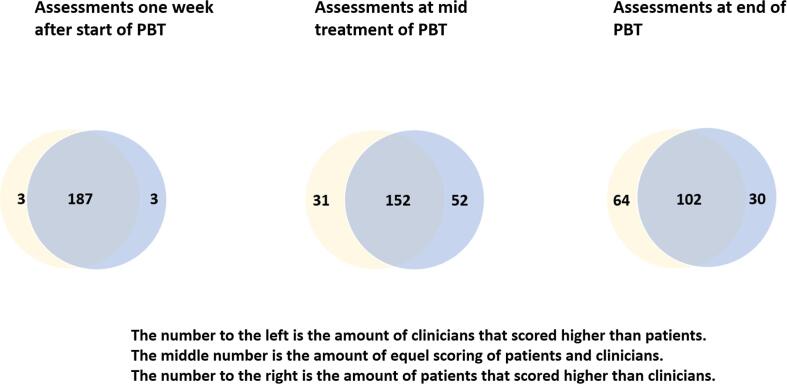
Agreement between the patients’ and clinicians’ assessments using the Radiotherapy-Related Symptom Assessment Scale (RSAS) intensity scores and the Radiation Therapy Oncology Group (RTOG) scores.

The MLR analysis explained 16% of RSAS variance. Three (1.2%) of 252 patients reported skin reactions and none reported distress from the skin reactions within one week after the start of PBT (Table 2). No associations were seen between patient-reported symptoms or clinician-reported signs of skin reactions and age, gender, education, occupation, other treatment or smoking. In 187 cases there were equal scoring of patients and clinicians (Fig. 1). At one week after start of PBT there was a significantly association between patient reported RSAS with higher total radiation dose (Table 3). The absolute agreement between patients’ and clinicians’ assessment was 97.3% and the kappa coefficients shows poor agreement (κ = −0.016) (Table 4).

**Table 2:** Distribution of patient-reported outcome from patients with primary brain tumour who underwent proton beam radiotherapy using the Radiotherapy-Related Symptom Assessment Scale (RSAS) scores for skin reactions frequency and intensity and symptom distress.

Skin reactions one week after start of PBT					
	RSAS intensity 1 = not at all, n (%)	RSAS intensity 2 = little, n (%)	RSAS intensity 3 = quite a bit, n (%)	RSAS intensity 4 = very much, n (%)	Total values
RSAS symptom distress 1 = no concern	249 (98.8)	3 (1.2)	0	0	252
RSAS symptom distress 2 = little concern	0	0	0	0	0
RSAS symptom distress 3 = quite a bit concern	0	0	0	0	0
RSAS symptom distress 4 = greatest concern	0	0	0	0	0
Total values	252	3	0	0	252

Skin reactions at mid treatment (3 weeks of PBT)					

	RSAS 1 = not at all, n (%)	RSAS 2 = little, n (%)	RSAS 3 = quite a bit, n (%)	RSAS 4 = very much, n (%)	Total values

RSAS symptom distress 1 = no concern	129 (35.6)	91 (15.8)	3(1.6)	0	223
RSAS symptom distress 2 = little concern	1 (11.1)	12 (25.7)	11 (4.0)	2 (1.2)	26
RSAS symptom distress 3 = quite a bit concern	0	0	0	0	0
RSAS symptom distress 4 = greatest concern	0	0	1	2	3
Total values	130	103	15	4	252

Skin reactions at end treatment (6 weeks of PBT)					

	RSAS 1 = not at all, n (%)	RSAS 2 = little, n (%)	RSAS 3 = quite a bit, n (%)	RSAS 4 = very much, n (%)	Total values

RSAS symptom distress 1 = no concern	115 (47.1)	82 (33.6)	5 (2.0)	0	202
RSAS symptom distress 2 = little concern	1 (0.4)	14 (5.7)	19 (7.8)	0	34
RSAS symptom distress 3 = quite a bit concern	0	0	5 (2.0)	0	5
RSAS symptom distress 4 = greatest concern	0	0	1 (0.4)	2 (0.8)	3
Total values	116	96	30	2	244

**Table 3:** Multiple linear regression analyses for the Radiotherapy-Related Symptom Assessment Scale (RSAS) and RTOG for patients with primary brain tumour who underwent proton beam radiotherapy (n = 253).

		Full model RSAS					Full model RTOG		
Dependent variable	Independent variable	B	95% CI for B	P	Dependent variable	Independent variable	B	95% CI for B	P
RSAS at one week after start of treatment		−0.093	−0.669–0.483	0.748	RTOG at timepoint 1			N/A	
	Age	0.002	0.000–0.005	0.102		Age			
	Sex	0.046	−0.005–0.096	0.079		Sex			
	Total radiation dose	0.016	0.006–0.025	0.001		Total radiation dose			
	Recidiv	0.028	−0.027–0.083	0.315		Recidiv			
	Other tumor treatment	−0.034	−0.133–0.065	0.495		Other tumor treatment			
	Education	0.036	−0.004–0.077	0.079		Education			
	Occupation	0.068	−0.013–0.149	0.100		Occupation			
	Smoking	−0.035	−0.110–0.041	0.362		Smoking			
	Model statistics	F (65) = 2.65, p 0.011, R^2^ = 0.16				Model statistics			

RSAS at mid treatment		−0.741	−4.534–3.051	0.698	RTOG at timepoint 2		−1.435	−4.452–1.583	0.344
	Age	−0.001	−0.170–0.160	0.931		Age	0.012	−0.001–0.026	0.077
	Sex	0.281	−0.540–0.617	0.099		Sex	−0.176	−0.462–0.111	0.224
	Total radiation dose	0.041	−0.210–0.103	0.189		Total radiation dose	0.057	0.008–0.107	0.025
	Recidiv	−0.053	−0.414–0.308	0.771		Recidiv	0.089	−0.225–0.404	0.570
	Other tumor treatment	−0.293	−0.945–0.359	0.372		Other tumor treatment	−0.410	−0.924–0.104	0.115
	Education	0.100	−0.169–0.369	0.460		Education	0.138	−0.120–0.396	0.288
	Occupation	−0.179	−0.716–0.357	0.507		Occupation	−0.327	−0.771–0.117	0.147
	Smoking	−0.004	−0.501–0.492	0.986		Smoking	0.003	−0.443–0.450	0.989
	Model statistics	F (65) = 1.16, p 0.332, R^2^ = 0.02				Model statistics	F (50) = 255.5, p 0.035, R^2^ = 0.16		

RSAS at end of treatment		−1.075	−4.759–2.610	0.562	RTOG at timepoint 3		−0.951	−4.551–2.649	0.598
	Age	−0.002	−0.018–0.014	0.785		Age	0.004	−0.013–0.021	0.621
	Sex	0.188	−0.138–0.514	0.255		Sex	−0.111	−0.466–0.243	0.532
	Total radiation dose	0.044	−0.017–0.104	0.152		Total radiation dose	0.040	−0.021–0.101	0.197
	Recidiv	−0.187	−0.538–0.164	0.291		Recidiv	−0.269	−0.650–0.112	0.163
	Other tumor treatment	−0.095	−0.728–0.538	0.766		Other tumor treatment	0.132	−0.598–0.861	0.719
	Education	0.143	−0.118–0.404	0.279		Education	0.187	−0.119–0.492	0.226
	Occupation	0.159	−0.362–0.681	0.544		Occupation	−0.096	−0.676–0.484	0.740
	Smoking	−0.049	−0.531–0.433	0.839		Smoking	0.198	−0.371–0.766	0.488
	Model statistics	F (65) = 0.74, p 0.668, R^2^ = -0.02				Model statistics	F (52) = 0.88, p 0.539, R^2^ = -0.02

**Table 4:** Agreements between patients’, with primary brain tumour who underwent proton beam radiotherapy, and clinicians’ assessments related to the presence of skin reactions symptoms through three time points (n = 253).

	Reactions reported by patient and missing report by oncologist; n (%)	Reactions reported by oncologist and missing report by the patient; n (%)	Reactions reported by both patient and oncologist; n (%)	Absolute agreement, %	Fleiss kappa	p-value
One week after start of PBT	59 (23.3)	1 (0.4)	193 (76.3)	96.9	−0.016	0.826
RTOG 0 – RSAS 1					−0.016	0.826
RTOG 1 – RSAS 2					−0.016	0.826

Mid treatment (3 weeks of PBT)	30 (11.9)	1 (0.4)	222 (87.7)	62.6	0.314	<0.001
RTOG 0 – RSAS 1					0.396	<0.001
RTOG 1 – RSAS 2					0.303	<0.001
RTOG 2 – RSAS 3					−0.045	0.505
RTOG 3–4 – RSAS 4					−0.009	0.892

End treatment (6 weeks of PBT)	52 (20.5)	5 (2.0)	196 (77.5)	52.0	0.214	<0.001
RTOG 0 – RSAS 1					0.296	<0.001
RTOG 1 – RSAS 2					0.151	0.034
RTOG 2 – RSAS 3					0.202	0.005
RTOG 3–4 – RSAS 4					−0.010	0.885

#### Skin reactions at mid treatment of PBT

At mid treatment of PBT there was a significant association between clinician’s reported RTOG with higher total radiation dose (Table 3). The MLR analysis explained 2% of the RSAS and 16% of the RTOG variance. One-hundred and twenty-two (48,4%) patients reported skin reactions and 26 (10.3%) reported distress from the skin reactions at mid treatment of PBT (Table 2). In 52 cases patients scored higher than clinician and in 31 cases clinicians scored higher than patients (Fig. 1). The absolute agreement between patients’ and oncologists’ assessment was 62.6% and the kappa coefficients range from poor (κ = −0.045) to moderate (κ = 0.396) agreement (Table 4).

#### Skin reactions at end treatment of PBT

At end of PBT there was no significant association between clinician’s reported RTOG or patient reported RSAS with any independent variables (Table 3). The MLR analysis explained −2% of the RSAS and −2% of the RTOG variance. According to the patient-reported skin reactions, 128 (52,4%) participants experienced skin reactions and 42 (17.2%) of the participants experienced concerns from the skin reactions at mid treatment of PBT (Table 2). In 30 cases patients scored higher than clinicians and in 64 cases clinicians scored higher than patients (Fig. 1). The absolute agreement between patients’ and oncologists’ assessment was 52.0% and the kappa coefficients range from poor (κ = −0.010) to moderate (κ = 0.296) agreement at end treatment of PBT (Table 4).

## Discussion

It is believed that this is the first study comparing patient reported skin reactions with clinicians’ reported skin reactions for patients diagnosed with primary brain tumour undergoing PBT treatment. This study showed a discrepancy between patient-reported and clinician-reported skin reactions and radiation dose was the only independent variable associated with increased skin reactions.

The results showed a significant association only for associations of skin reactions and radiation dose with a statistical significance level at p < 0.05. This is well known and expected as higher cumulative radiation dose increases the presence of skin reaction [8]. It is a challenge to reduce the radiation dose administrated without compromising the effect of the treatment [29]. There is no strong evidence for how to prevent skin reactions [29]. It may be more important to prevent skin reaction for patients undergoing PBT due to a possible higher risk for skin reactions compared to photon therapy [12]. A low number of smokers (13,8%) may explain no correlation to skin reactions wich is not in line with earlier studies [30], [31], [32]. Age [30], [31] or gender [8] did not seem to play a role in skin reactions which is in agreement with earlier studies.

The results showed a discrepancy between the PRO and CRO with poor to moderate kappa scores agreement. Discrepancy between the PROs and CRO is consistent with earlier studies [21], [33], which could be explained by several factors, e.g. different criteria are considered in the assessment due to varying of patients’ and clinicians’ perspectives [34], [35]. This may be due to skin reactions consisting of both observable signs (e.g. erythema) and non-observable symptoms (e.g. itching), which suggests that patients and clinicians are not reporting the same thing. Previous studies have shown that agreement tends to be higher for observable signs compared to non-observable symptoms [33], [34], [35], [36], [37]. Symptoms may appear earlier than signs. Therefore, the patient could report symptoms earlier than the clinician, which is in line with earlier studies [34], [35]. Kirchheiner et al [38] argued that some discrepancy between PRO and CRO may be acceptable due to methodological differences.

Moreover, clinicians are at high risk of under-reporting subjective toxicities within randomized trials [21]. The most common discrepancy found in earlier studies had been clinicians’ under-reporting, but in the present study it was also shown that after six weeks of PBT 22% of the patients did not report skin reactions while their clinicians did so. Which may be explained by the clinicians are extra observant in reporting symptoms or may be that patients expectations and their fear for skin reactions may decrease their perceptions [39]. It could also be that clinicians are more sensitive to largely expected symptoms [21]. Another explanation could be that patients may believe that less severe symptoms are not that relevant to be reported, especially if they experience other, more severe symptoms that are worse or that they expected skin reactions to appear.

Furthermore, at the end of treatment, the clinicians reported the presence of skin reactions (any grade) for 71% of the patients, while only 53% of the patients reported skin reactions (any grade) at the same time point. This was an unexpected result because the values were lower than one earlier study that showed 86% of the patients reported experiencing some grade of acute skin toxicity [8], which may be explained by the newer PBS technique compared to the passive scatter technique.

We believe that reporting skin reactions only by clinicians may not be sufficiently accurate. PROs provide an opportunity to understand the patients’ own perceptions [13]. The findings in this study support the incorporation of PROs into reporting of skin reactions among patients undergoing PBT treatment and other radiotherapy modalities, and that—combined with clinicians’ assessments—they will enhance the probabilities of achieving the best path for patients’ symptoms relief. Implementing PROs is a way to increase the patient’s involvement in their care, where their unique needs are accounted for when determining suitable interventions. On the other side, the newly identified discrepancy might make it more difficult to interpret these two assessments. Since the assessments are not comparable with each other and have slightly different targets.

There are strengths and limitations of this study. One strength was examining skin reactions that are a common symptom but often not the worst symptom during the treatment period and therefore not frequently included in investigations. Methodologically, RTOG and RSAS may not be comparable because of their own scoring and different categorisations. One limitation was that baseline data were collected one week after starting the PBT because it is rare to have developed skin reactions from PBT during the first week. Another limitation was that no follow up data three weeks after finished the PBT were included. Likewise, it was a limitation to not include more variables in the analysis, e.g. hair loss or capture more qualitative data.

## Conclusions

There was a poor agreement between PRO and CRO skin reactions. This shows that the patient needs to be involved in assessments of skin reactions to be able to get a more complete understanding of skin reactions due to PBT. This may also improve patient experience regarding involvement in their own care. To better understand skin reactions, it is important to incorporate both PROs and CROs. Further studies are needed to explore how and when it is best to use PROs or CROs alone and when to combined PROs and CROs. Furthermore, it is also needed to receive a better understanding in how clinicians can interpret and understand the PROs as a part of their working tool. The long-term ambition is that PROs can give a complete information about the presence, intensity and experiences of the toxicity during and after cancer treatment.

## Compliance with ethical standards

This multicentre study has been approved by the research ethic committee (2015-07-22, Dnr:433-15). Informed consent was obtained from all individual participants included in the study.

## Funding

This research was funded by grants (CAN2015/428 and CAN2016/809) from the 10.13039/100012538Swedish Cancer Foundation.

## Declaration of Competing Interest

The authors declare that they have no known competing financial interests or personal relationships that could have appeared to influence the work reported in this paper.
